# Sport event destination image promotes subsequent sport participation through sport attraction: the moderating role of perceived stress

**DOI:** 10.3389/fpsyg.2026.1863652

**Published:** 2026-08-03

**Authors:** Danping Liu, Jing Liu, Ke Yang, Di Hu, Jingchen Ma, Hanjia Ran, Yimin Song

**Affiliations:** 1 School of Management, Xihua University, Chengdu, China; 2 School of Financial Management, Gingko College of Hospitality Management, Chengdu, China; 3 Sichuan Sports College, Chengdu, China; 4 Academy of Art and Design, Anhui University of Technology, Maanshan, China

**Keywords:** perceived stress, psychological continuum model, sport attraction, sport event destination image, sport event tourism, sport participation

## Abstract

**Background:**

Drawing on the psychological continuum model and spillover effect theory, this study examines whether and how sport event destination image promotes young tourists’ near-term post-event sport participation through sport attraction, and whether this relationship is contingent on perceived stress. While prior research has suggested that sport event destination image is positively associated with post-event participation and strengthens sport attraction, the underlying mechanisms and moderating conditions remain insufficiently integrated.

**Methods:**

We used a two-wave survey design among Chinese tourists who attended the 31st FISU World University Games in Chengdu as spectators. Data were collected at two time points with a one-month interval, yielding 290 valid matched responses. The moderated mediation model was tested using partial least squares structural equation modeling.

**Results:**

Sport event destination image positively predicted near-term post-event sport participation. Sport event destination image also positively affected sport attraction, which in turn positively influenced near-term post-event sport participation. In addition, sport attraction mediated the relationship between sport event destination image and near-term post-event sport participation. Perceived stress significantly and negatively moderated the association between sport attraction and near-term post-event sport participation, such that the positive effect of sport attraction was weaker among individuals with higher perceived stress.

**Discussion:**

This study extends destination image research by demonstrating the behavioral spillover effect of sport event destination image on near-term post-event sport participation. It also advances the application of the psychological continuum model in sport tourism by identifying sport attraction as a key psychological mechanism and perceived stress as an important boundary condition. From a practical perspective, the findings suggest that sport event destinations can foster near-term post-event sport participation not only by strengthening destination image and sport attraction, but also by addressing tourists’ perceived stress after the event.

## 1 Introduction

Sport tourism, a rapidly expanding sector at the intersection of tourism and sport, refers to leisure-related travel for participating in physical activities, attending sporting events, and visiting sport-related attractions (Gibson, 1998). Sport event tourism is an important subcategory of sport tourism and involves travel motivated by attendance at sporting events, attracting visitors from around the world to events such as the Olympic Games (Deery et al., 2004). Hosting major sporting events can enhance urban visibility, attract tourists, and generate economic benefits (Taks et al., 2014). Consequently, many cities have adopted various strategies to compete for the opportunity to host such events.

Destination image has long been recognized as important in the tourism literature. Duan and Wu (2024) found that destination image affects visitors’ revisit intentions, whereas Wei et al. (2024) demonstrated its influence on destination attachment. King et al. (2015) further showed that destination image may fade over time after an event. In the sport context, Thomson et al. (2019) observed increased sport participation among individuals exposed to the Olympic Games, and Chalip et al. (2017) suggested that integrating events into sport marketing strategies may promote sport participation. Despite this growing body of research, the relationship between destination image and near-term post-event sport participation remains insufficiently understood. Although sport participation is a central topic in social behavior research, previous studies have mainly focused on visitors’ behavioral responses during sport tourism experiences, while paying limited attention to behavioral changes after the visit. This gap motivates the present study, which seeks to examine whether and how destination image influences near-term post-event sport participation.

Previous research has shown that destination image shapes visitors’ psychological evaluations, such as satisfaction, attachment, and loyalty, and that favorable evaluations enhance destination attractiveness and stimulate recommendation and revisit intentions (Afshardoost and Eshaghi, 2020; Pereira et al., 2022). According to spillover effect theory (Googins, 1991), sport-related attitudes and experiences developed during tourism may transfer to individuals’ everyday lives, suggesting that sport attraction generated in a tourism context may encourage near-term post-event sport participation. In addition, scholars have identified sport attraction as an important psychological antecedent of sustained sport participation (Baker et al., 2020). Extending this logic to sport tourism and drawing on the psychological continuum model (PCM), we propose that destination image may strengthen sport attraction, which in turn promotes sport participation. However, this mechanism is likely to depend on perceived stress, given its negative association with engagement in physical activity (Martín-Rodríguez et al., 2024). Stress and emotion are closely intertwined (Lazarus, 1999, 2000), and tourism experiences have been shown to affect perceived stress in multiple ways (Jordan and Vogt, 2017). Positive tourism experiences, in particular, may reduce tourists’ perceived stress (Zhu et al., 2020). Based on these arguments, we propose a moderated mediation model grounded in the PCM, in which destination image influences sport participation through sport attraction, while perceived stress attenuates the effect of sport attraction on sport participation.

A better understanding of how destination image influences sport participation may help governments, sport management organizations, and destination and venue managers develop more effective marketing strategies that address visitors’ travel needs, promote tourism development, encourage engagement in sport, and ultimately improve public health. Drawing on the psychological continuum model and spillover effect theory, this study develops a theoretical framework linking sport event destination image, sport attraction, sport participation, and perceived stress. Specifically, the present research seeks to clarify how and under what conditions destination image influences tourists’ near-term post-event sport participation.

## 2 Literature review and hypothesis development

### 2.1 The psychological continuum model

From sociological and psychological perspectives, the PCM posits that individuals progress through four stages of psychological development—awareness, attraction, attachment, and allegiance—shaped by both external and internal factors (Funk and James, 2001). In the present study, the PCM provides a useful framework for understanding how psychological development may shape sport participation (Funk and James, 2001). Zhang et al. (2019) argued that destination image reflects the cognitive and affective evaluations individuals form throughout these stages of psychological progression. At the awareness stage, individuals acquire basic knowledge of destinations, events, and sports, although they may not yet develop strong personal interest. As they gain further exposure and information, they enter the attraction stage and begin to develop preferences and favorable attitudes (Beaton et al., 2009). Recognizing this process, destination marketers often seek to highlight and promote destination attributes in order to stimulate such attraction (Chen and Funk, 2010). From both tourism marketing and individual psychological perspectives, destination image therefore plays an important role in drawing individuals toward sport events and sport-related activities. At the attachment stage, individuals develop a relatively stable psychological bond with the destination, event, or sport. Finally, at the allegiance stage, this bond becomes integrated into their core values and beliefs, leading them to engage more actively and consistently in related activities.

Sport event tourism is typically brief in duration, meaning that tourists are likely to remain in the attraction stage during the trip itself. We argue, however, that this stage may serve as a critical starting point for subsequent behavioral development. Specifically, individuals with stronger sport-related interest may continue to expand their sport knowledge and participation after the trip, gradually forming a habit of active engagement. As this process unfolds, their psychological orientation may progress from attraction to attachment and eventually to allegiance. Accordingly, this study adopts the attraction-stage logic of the PCM to explain near-term post-event sport participation, with particular emphasis on the roles of destination image and sport attraction in this process.

### 2.2 Destination image

Hunt (1971) described destination image as individuals’ perceptions of places in which they do not reside. Baud-Bovy and Lawson (1977) further explained that destination image comprises visitors’ overall knowledge, impressions, prejudices, imaginings, and emotional thoughts about a locale or place. Tourists often evaluate a destination based on how their perceived destination image in tourism section (Yang et al., 2022a). Over the years, scholars have continued to refine this concept. Destination image evolves dynamically through the accumulation of information (Gunn, 1988) and is shaped by intrinsic factors, such as motivations and risk perceptions, as well as external stimuli, such as marketing campaigns and experiential encounters. Agapito et al. (2013) demonstrated that a destination’s image influences visitors’ behavior across three distinct phases: before, during, and after the visit. As discussed earlier in relation to the PCM, destination image is one of the key factors that attract tourists and lays the foundation for their progression to the attachment stage and subsequent phases.

The concept of destination image is multifaceted and includes attributes such as multiplicity, complexity, relativism, and dynamism (Gallarza et al., 2002). Nonetheless, most researchers approach destination image as a multi-attribute construct. A two-dimensional model comprising cognitive and affective images was proposed by Baloglu and McCleary (1999). Repeated interactions increase the complexity of destination image and foster more favorable perceptions among repeat visitors (Konečnik and Ruzzier, 2006; Yang et al., 2022c, 2021, 2025a). Several scholars have highlighted that the main conceptual frameworks of destination image, including the three-dimensional continuum approach and the three-component approach, both recognize the cognitive and affective elements of destination image formation and their influence on tourists’ behavioral responses (Zhang and Yun, 2014; Yang et al., 2022b). This study mainly adopts a two-dimensional model of destination image that includes cognitive and affective images.

The attractions that shape a destination’s image include nature-based attractions (Hao et al., 2025), cultural heritage tourism (Zhou et al., 2023), and food tourism (Cifci et al., 2024). In addition to these attractions, sports events can also enhance a destination’s image and contribute to increased visitor flows (Morgan et al., 2021). This effect is particularly evident when local sporting events are well organized and effectively publicized, as they can make the destination more attractive to potential tourists. In contrast, poor event organization and negative media coverage may damage a destination’s image and reduce its appeal (Mamo et al., 2026). Therefore, building a positive destination image requires sustained sports marketing efforts and the effective integration of sporting events into broader tourism activities. In this regard, sporting events play a significant role in tourism marketing by providing host destinations with a powerful platform to showcase their attractiveness and distinctive image to visitors (Jia et al., 2025). Supporting this view, Bazzanella et al. (2023), based on a systematic review, argued that sports events function as strategic assets for host destinations by enhancing destination visibility, stimulating economic development, and strengthening competitive positioning in the global tourism market. Furthermore, their findings suggest that a strong destination image can attract new market segments and expand tourism demand. In the context of the present study, destination image is conceptualized as tourists’ cognitive and affective perceptions and overall impressions of a sport event destination. Specifically, it encompasses perceptions of infrastructure, accessibility, cultural attractions, crowding, and the extent to which the destination provides a joyful and enjoyable experience (De Los Reyes and Dael, 2023). Accordingly, destination image is incorporated as an antecedent variable in the proposed research model.

### 2.3 Sport participation

Sport participation is a multidimensional construct that encompasses behavioral, cognitive, and affective domains, including direct engagement, media consumption, social discussion, knowledge acquisition, and emotional investment (Snyder and Spreitzer, 1983). Existing research on sport participation has primarily focused on its implications for individuals and society. At the individual level, one of the most widely recognized benefits of sport participation is its contribution to physical health and overall well-being (Silva et al., 2020). In addition, sport participation has been associated with reduced negative psychological states, such as depression, anxiety, and stress. Recent studies have further suggested that sport participation may help reduce violent tendencies and hooligan behaviors (Nickodem et al., 2023). Muñoz-Bullón et al. (2017), for example, identified a positive association between college students’ sport participation and academic performance. Similarly, Yan et al. (2021) found that children who participated in team sports tended to report stronger athletic self-concepts and higher self-esteem. At the societal level, sport participation has been linked to increased consumption of sport-related products and broader economic and social development (Pye et al., 2015). Overall, previous research has consistently portrayed sport participation as a set of broadly beneficial behaviors.

Prior studies have identified a range of factors influencing individual sport participation, including gender, socioeconomic status, sociodemographic characteristics, proximity to exercise facilities, family physical culture (Strandbu et al., 2020), prior physical experience (Scicluna et al., 2023), parental and partner involvement, and school physical education programs. In the context of tourism, scholars such as Dickson et al. (2021) have argued that hosting mega-sport events may promote sport participation and contribute to the development of a sport tourism legacy. Given the importance of understanding the factors that encourage sport participation, the present study treats near-term post-event sport participation as the dependent variable and examines the mechanism linking destination image to this outcome.

### 2.4 Hypothesis development

#### 2.4.1 Destination image and sport participation

Trotier (2017) argued that hosting multiple sporting events can contribute to branding a city as a “sports city,” thereby increasing visitors’ willingness to engage in and support future events. Chen and Funk (2010) further observed that sport tourists tend to spend more on sport-related activities than on other types of attractions, suggesting a stronger inclination toward sport involvement. Given that sport participation is responsive to environmental changes (Fredricks et al., 2004), it is reasonable to expect that it may also be influenced by destination image. A substantial body of research has confirmed the important role of destination image in shaping visitors’ behavioral intentions. Because intention is widely recognized as a key predictor of behavior (Webb and Sheeran, 2006), we extend this reasoning to suggest that the image of the host city may significantly influence tourists’ sport-related behaviors after attending a sporting event. From the perspective of the psychological continuum model (PCM), activity involvement can be understood as a process in which inputs shape psychological processes and ultimately lead to behavioral outcomes (Beaton et al., 2011). In this context, tourists’ perceptions of destination image may serve as an important input that influences their psychological responses and encourages near-term post-event sport participation (Hungenberg et al., 2016), thereby fostering greater understanding of and engagement in sport. Moreover, in line with the PCM, individuals who progress to the allegiance stage are more likely to demonstrate strong advocacy, immediate participation, and other subsequent sport-related behaviors. Accordingly, we propose that a positive destination image may promote tourists’ near-term post-event sport participation.

*H1:* Sport event destination image has a positive effect on near-term post-event sport participation.

#### 2.4.2 Destination image and sport attraction

The Oxford English Dictionary defines attraction as a feature, quality, or person that makes something seem interesting, enjoyable, or worth having or doing. Drawing on this definition, as well as related concepts such as human–machine attraction and other forms of attraction, McIntyre and Pigram (1992) defined sport attraction as an individual’s evaluation of and enjoyment derived from sport. Sport attraction represents an important stage in an individual’s psychological progression toward dependence on sport and is also shaped by psychosocial factors and hedonic motivation (Funk and James, 2001). Accordingly, fostering individuals’ attraction to sport is essential for the development of sport (Sotiriadou et al., 2008).

Sport event tourism capitalizes on the attributes of both sport and destination to attract tourists (Taks et al., 2014). Before an event, host cities often seek to enhance their image by promoting sport-related knowledge and local culture in order to attract potential visitors. A positive destination image of a sport event venue can attract not only tourists who are already passionate about sport, but also those who are interested in watching sporting performances (Li et al., 2023). Moreover, a favorable destination image may also engage travelers who might not otherwise consider attending a sporting event. Based on this reasoning, we propose the following hypothesis:

*H2:* Sport event destination image has a positive effect on sport attraction.

#### 2.4.3 Sport attraction and sport participation

Researchers have identified outcome uncertainty, athletic display, the kinesthetic nature of sport activities, and the visceral character of many forms of sport engagement as key features of sport attraction (Hinch and Higham, 2005). These elements contribute substantially to the enjoyment experienced by sport participants. Sport attraction not only enhances individuals’ awareness of physical activity but also stimulates greater participation in sport (Funk, 2017). This appeal is particularly important for adolescents, as it may encourage them to join sport clubs and potentially foster the development of future elite athletes (Grix and Carmichael, 2012). Furthermore, individuals who are attracted to sport often invest considerable resources, including time, money, and emotional energy, which can lead to the formation of psychological bonds with sports, athletes, or sport-related groups (Liu et al., 2021). Over time, such investment may develop into deeper attachment and manifest as fandom for one or more sports. Although the focus of attraction may differ across contexts, research in organizational behavior has shown that attractiveness positively influences job seekers’ decision-making (Highhouse et al., 2003), offering useful insights for understanding similar dynamics in the sport domain. Accordingly, we propose the following hypothesis:

*H3:* Sport attraction has a positive effect on near-term post-event sport participation.

#### 2.4.4 The mediating role of sport attraction

The PCM posits that attraction represents a critical phase in psychological continuity that is necessary for behavior to emerge (Chen and Funk, 2010). As a psychological state, attraction emerges when individuals form specific perceptions, emotional responses, and positive evaluations toward an object or activity (Byrne, 1997). In the context of sport event tourism, tourists’ perceptions and evaluations of destination image may activate sport attraction. This attraction reflects a transitional dynamic from the attraction stage to the attachment stage, thereby facilitating the formation of attitudinal preferences. Previous research has demonstrated that destination image is associated with the subsequent development of attachment and loyalty, both of which are linked to stronger attraction and behavioral change (Stylidis et al., 2020). Accordingly, we propose that sport attraction may mediate the relationship between destination image and visitors’ near-term post-event sport participation.

*H4:* Sport attraction mediates the relationship between sport event destination image and near-term post-event sport participation.

#### 2.4.5 The moderating role of perceived stress

Stress, as defined by McEwen (2007), refers to the experience of emotional and physical challenges. Perceived stress, in turn, denotes the degree to which individuals evaluate situations in their lives as stressful (Cohen et al., 1983). It reflects an imbalanced state in which individuals feel threatened by internal or external sources (Chrousos and Gold, 1992). The perception of stress depends on how individuals appraise potential stressors as threatening or non-threatening, as well as on their perceived capacity to cope with them (Liu et al., 2021). A range of factors may influence stress levels. Research has shown that active participation in physical activity is associated with lower levels of stress (Stanton et al., 2020). Lee and Vincent (2021) further suggested that self-concept clarity can reduce perceived stress and mediate the relationship between sport participation and stress. Personality traits have also been found to shape stress levels (Liu et al., 2021). In addition, scholars have identified stress as a factor that directly affects exercise behavior (Dunker et al., 2020). At the same time, leisure travel is often regarded as an effective means of enhancing well-being and relieving stress (Zins and Ponocny, 2022). After a trip, however, returning to everyday life may also be accompanied by changes in stress levels (Yu et al., 2021).

Stress levels are closely related to individuals’ emotions, and these emotions ultimately influence what people do and how they behave (Russell and Mehrabian, 1975). Previous studies have shown that tourism experiences can significantly affect tourists’ perceived stress (Jordan and Vogt, 2017), whereas positive emotions can enhance tourists’ engagement behaviors (Goossens, 2000; Baker and Crompton, 2000; Bigné et al., 2005). Extending this logic to sport tourism, and drawing on the PCM, we argue that perceived stress may shape the extent to which sport attraction translates into actual sport participation. Specifically, because stress may constrain individuals’ motivation, energy, and willingness to remain physically active, higher levels of perceived stress may weaken the positive relationship between sport attraction and near-term post-event sport participation. Accordingly, we propose that perceived stress moderates the relationship between sport attraction and near-term post-event sport participation. In this study, perceived stress is therefore introduced as a boundary condition to examine the limits of the effect of sport attraction.

Based on this, we hypothesize that:

*H5:* Perceived stress moderates the relationship between sport attraction and near-term post-event sport participation.

**Figure 1:**
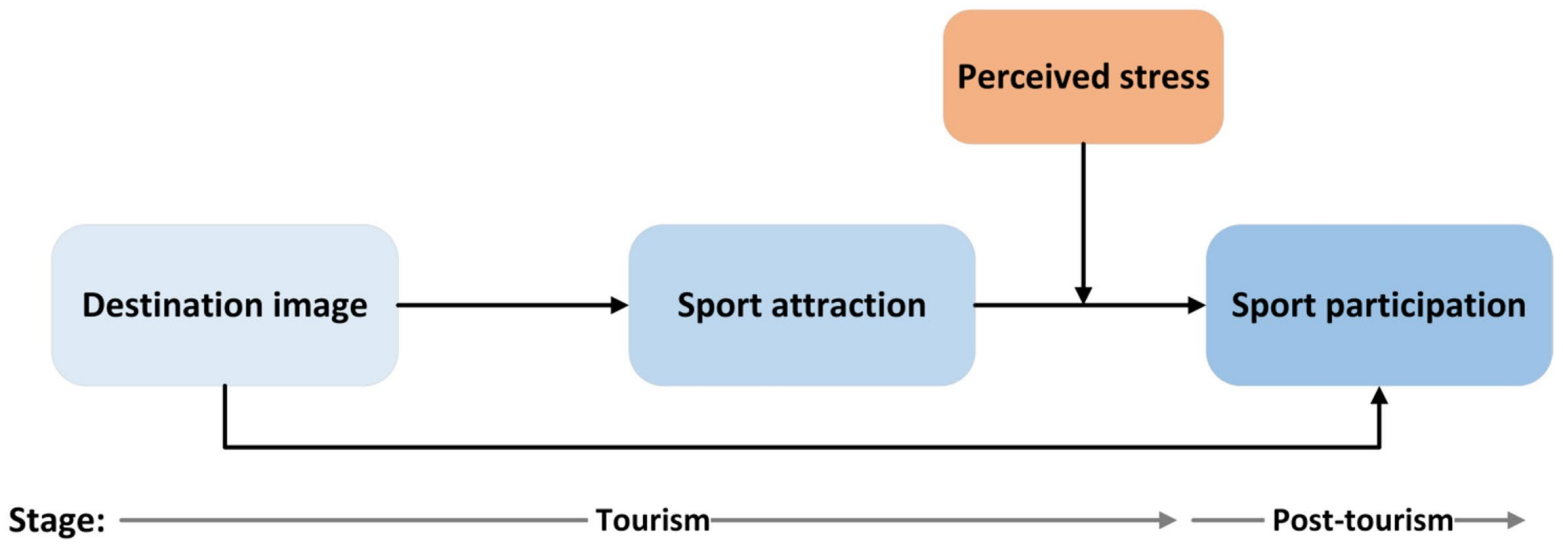
The conceptual model.

Based on this analysis, Figure 1 depicts the study’s conceptual model.

## 3 Method

### 3.1 Samples and procedures

In this study, we examined the impact of sport event destination image on tourists’ near-term post-event sport participation. In recent years, many Chinese cities have increasingly recognized the economic and social benefits associated with hosting sporting events. Chengdu, for example, has actively promoted itself as a “city of sport events” and has gained international recognition by hosting major competitions. As one of China’s premier tourist destinations, Chengdu is home to the 3,000-year-old Jinsha Site and is widely recognized as the birthplace of ancient Shu civilization. The city was scheduled to host five major international sporting events between 2019 and 2025, in addition to several smaller events. The 31st FISU World University Games, held in Chengdu in August 2023, therefore provided a valuable opportunity for data collection.

This study targeted Chinese tourists who attended the 31st FISU World University Games in Chengdu primarily as spectators. Since most tourists attending the event were young college students, this study focused primarily on young student tourists. To enhance data accuracy, participants were randomly recruited across 27 venues. The research was conducted in two phases separated by a one-month interval. In the first phase, conducted in August 2023, 623 visitors were invited to join a WeChat group with the chat function disabled and to complete the first questionnaire. Each respondent was assigned a unique four-digit code to match responses across the two survey waves while ensuring confidentiality. To ensure data accuracy and mitigate common method bias, the two surveys were conducted one month apart using identical questionnaires. In the second phase, 307 responses were collected through the same WeChat group, yielding a response rate of 49.28%. Of these, 290 questionnaires were valid, resulting in an effective rate of 94.46%. In terms of demographics, respondents were predominantly female (63.1%), aged 18–25 years (88.3%), students (87.2%), and low-income earners, with 83.8% reporting a monthly income below 3,000 RMB; 80.3% held a bachelor’s degree. Detailed sample characteristics are presented in Table 1.

**Table 1:** Sample profile (*n* = 290).

Variable	Item	*n*	%
Gender	Male	107	36.9
	Female	183	63.1
Age	Below 18 years	4	1.4
	18–25 years	256	88.3
	26–30 years	4	1.4
	31–40 years	15	5.2
	41–50 years	10	3.4
	51–60 years	0	0.0
	More than 60 years	1	0.3
Job	Student	253	87.2
	Government and public institutions employ	12	4.1
	Freelancer	6	2.1
	Employees of the private sector	8	2.8
	Self-employed person	4	1.4
	Others	7	2.4
Income	Less than 3,000 RMB	243	83.8
	3,001–6,000 RMB	27	9.3
	6,001–9,000 RMB	11	3.8
	More than 9,000 RMB	9	3.1
Marriage	Married	22	7.6
	Divorced	1	0.3
	Single	212	73.1
	Others	55	19.0
Education	High school and below	22	7.6
	Junior college	233	80.3
	Bachelor’s degree	29	10.0
	Master’s degree	2	0.7
	Doctoral degree	4	1.4

### 3.2 Measurement

The study employed a structured questionnaire consisting of five sections: (1) destination image, (2) sport attraction, (3) perceived stress, (4) sport participation, and (5) respondents’ demographic characteristics. The questionnaire was initially developed in English and then translated into Chinese by bilingual experts following Brislin’s (1980) back-translation procedure to ensure translation accuracy and correct any inconsistencies. All items were measured on a five-point Likert scale ranging from 1 (strongly disagree) to 5 (strongly agree).

We used multi-item reflective measures that had been validated in previous research. The destination image section comprised 10 items adapted from Ekinci and Hosany (2006) to assess tourists’ cognitive and affective evaluations of Chengdu’s image as a sport event destination. Sport attraction was measured using a four-item scale adapted from Kyle and Mowen (2005). Tourists’ perceived stress was assessed using the Perceived Stress Scale (PSS) developed by Cohen (1994), including items such as “In the last month, how often have you been upset because of something that happened unexpectedly?” (1 = not at all; 5 = very often). Sport participation was measured using the eight-item scale developed by Snyder and Spreitzer (1983), which has demonstrated broad applicability across previous studies. Given the short interval between the two rounds of questionnaire distribution, this study mainly focused on measuring near-term post-event sport participation. In addition, to account for potential differences in perceptions of destination image and perceived stress across demographic groups, we controlled for gender, age, income, education, occupation, and marital status.

### 3.3 Pre-test

The study employed established scales. To assess the validity and applicability of the questionnaire (Podsakoff et al., 2003), a pilot test was conducted using a subset of the target sample. In the first round, 103 questionnaires were distributed, and 85 valid responses were obtained, yielding a response rate of 82.5%. In the second round, 85 questionnaires were distributed, and 66 responses were collected, corresponding to a response rate of 77.2%.

Reliability was primarily assessed using Cronbach’s alpha, which measures the internal consistency of a scale and is generally expected to exceed 0.70 (Bland and Altman, 1986). In addition, the corrected item–total correlation (CITC) was used to determine whether item deletion would improve reliability, with 0.40 adopted as the minimum acceptable threshold (Farh et al., 1997). SPSS analysis showed that the Cronbach’s alpha coefficients for destination image, sport attraction, sport participation, and perceived stress were 0.811, 0.887, 0.848, and 0.920, respectively. However, items Q9 and Q10 in the destination image scale, Q6 in the sport participation scale, and Q4 and Q9 in the perceived stress scale had CITC values below 0.40 and were therefore removed. The reliability and validity results for the refined sample are reported in Table 2.

**Table 2:** The reliability and validity of the refined sample.

Time	Variable		Cronbach α		KMO	
T1	Destination image	Affection image	0.975	0.820	0.613	0.835
		Cognition image	0.965		0.676	
	Sport attraction		0.893		0.721	
T2	Sport participation	Affection participation	0.893	0.780	0.852	0.796
		Cognition participation	0.742		0.5	
	Perceived stress		0.931		0.804	

### 3.4 Analytic technique

In this study, partial least squares structural equation modeling (PLS-SEM) was employed to examine the causal relationships among the core study variables. PLS-SEM was deemed particularly appropriate for three key reasons. First, it does not assume normally distributed data (Hair et al., 2021). Second, this analytical tool is not only well established across the social sciences but is also extensively used in the marketing and hospitality field, especially for analyzing complex research models (Yang et al., 2025b; Zhu et al., 2026; Fan et al., 2023). We adopted SmartPLS 4 to implement the PLS-SEM procedure, which can effectively accommodate the structural characteristics of the research data and enhance the robustness of the model estimation results.

The proposed model comprised 28 observable indicators and five latent variables, including two constructs related to destination image and three related to near-term post-event sport participation. Following the recommendations of Hair et al. (2011), the complete PLS-SEM analytical procedure was divided into two core stages: evaluation of the measurement model and evaluation of the structural model. In the measurement model stage, we systematically tested the internal consistency reliability and construct validity of all latent variables to ensure that the designed scale demonstrated adequate stability and validity. In the structural model stage, we further examined the path relationships, coefficient significance, and explanatory power among latent variables. Subsequently, the significance of the hypothesized relationships specified in the structural model was quantitatively examined. All research hypotheses were formally tested using the standard PLS algorithm and a bootstrapping procedure with 5,000 resamples, as recommended by Hair et al. (2014), which helped reduce the influence of random sampling error and ensured the statistical robustness of the hypothesis test results.

## 4 Results

### 4.1 Common method variance

We performed a statistical test for the potential effect of common method variance (CMV) using Harman’s one-factor test (Podsakoff et al., 2003). Principal factor analysis with Varimax rotation was performed to determine whether a single method factor explained most of the variance. More than one factor with an eigenvalue greater than 1 was extracted, and the first factor explained 28.27% of the total variance. Therefore, CMV did not appear to be a serious problem in this study.

### 4.2 Measurement model assessment

Prior to assessing the structural model, it is necessary to establish the reliability and validity of the measurement model. As suggested, factor loadings should ideally exceed 0.6, while items with factor loadings below 0.5 are typically candidates for removal (Hair et al., 2017; Hair et al., 2019). For construct validity, particularly convergent validity, composite reliability (CR) and average variance extracted (AVE) values should exceed 0.7 and 0.6, respectively (Fornell and Larcker, 1981). As shown in Table 3, the measurement model analysis indicated that all items had factor loadings above 0.6. The AVE values ranged from 0.690 to 0.904, while the CR values for all constructs exceeded 0.8, supporting the reliability and convergent validity of the model. As shown in Table 4, discriminant validity was established, with HTMT ratio values below the conservative threshold of 0.90 (Henseler et al., 2015).

**Table 3:** Measurement model.

Item	Mean	S.D.	Loading	KMO	Alpha	CR	AVE
Destination image				0.860	0.879	0.987	0.901
Affection image				0.843	0.961	0.973	0.898
Q1	4.72	0.640	0.929				
Q2	4.74	0.597	0.958				
Q3	4.74	0.592	0.943				
Q4	4.74	0.597	0.961				
Cognition image				0.876	0.964	0.974	0.904
Q5	4.34	0.867	0.935				
Q6	4.39	0.882	0.947				
Q7	4.41	0.837	0.960				
Q8	4.38	0.865	0.961				
Sport attraction				0.832	0.923	0.946	0.814
Q9	4.34	0.797	0.819				
Q10	4.24	0.827	0.942				
Q11	4.03	0.930	0.922				
Q12	4.15	0.906	0.920				
Perceived stress				0.888	0.934	0.947	0.690
Q13	4.25	0.791	0.818				
Q14	4.27	0.810	0.824				
Q15	4.25	0.789	0.842				
Q16	3.97	0.950	0.842				
Q17	3.94	0.970	0.885				
Q18	3.72	1.029	0.813				
Q19	4.17	0.772	0.829				
Q20	4.3	0.703	0.791				
Sport participation				0.819	0.741	0.962	0.788
Affection participation				0.878	0.911	0.939	0.759
Q21	3.31	1.300	0.600				
Q22	3.46	1.135	0.915				
Q23	3.36	1.186	0.941				
Q24	3.23	1.231	0.936				
Q25	3.28	1.237	0.915				
Cognition participation				0.500	0.835	0.924	0.859
Q26	4.07	1.235	0.927				
Q27	3.82	1.314	0.927				
Behavioral participation							
Q28	3.49	0.777					

**Table 4:** Discriminant validity (heterotrait–monotrait [HTMT] ratios).

Variables	Destination image	Sport participation	Sport attraction	Perceived stress
Destination image				
Sport participation	0.226			
Sport attraction	0.318	0.677		
Perceived stress	0.267	0.568	0.699	

### 4.3 Structural model and hypothesis testing

R^2^ estimates standardized path coefficients (*β*) and significance levels (t-values). The R^2^ values for the endogenous variables—sport attraction and sport participation—were 0.118 and 0.462, respectively, both exceeding the recommended threshold of 0.10 (Leguina, 2015).

We employed a blindfolding procedure to evaluate the model’s predictive relevance using the Q^2^ statistic (Leguina, 2015). The predictive relevance of the model was confirmed by the Q^2^ values of the endogenous constructs, which were 0.501 for destination image, 0.759 for sport attraction, 0.654 for perceived stress, and 0.434 for sport participation. As all values exceeded zero, the model demonstrated acceptable predictive relevance.

Table 4 presents the variance inflation factor (VIF) and Cohen’s F^2^ effect sizes for all hypothesized paths. The VIF values for all paths range from 1.000 to 1.927, which are well below the conservative threshold of 3, indicating that multicollinearity is not a concern (Hair et al., 2019).

**Figure 2:**
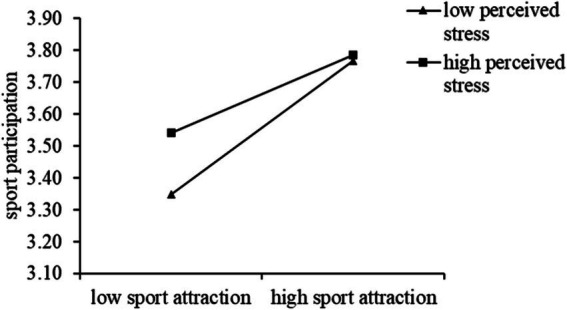
The interaction between sport attraction and perceived stress on tourists’ sport participation.

PLS-SEM was employed in this study to examine the relationships among the variables and test the proposed hypotheses. Hypothesis evaluation was primarily based on path coefficients, standard errors, and *p*-values. As shown in Table 5, the results indicated that the hypothesized relationships among destination image, sport attraction, near-term post-event sport participation, and perceived stress were generally supported. Specifically, destination image had significant positive effects on both near-term post-event sport participation and sport attraction (*β* = 0.192, *p* < 0.01, SD = 0.058; *β* = 0.333, *p* < 0.001, SD = 0.095), thereby supporting H1 and H2. Sport attraction also had a significant positive effect on near-term post-event sport participation (*β* = 0.241, *p* < 0.005, SD = 0.027), supporting H3. Furthermore, sport attraction partially mediated the relationship between destination image and near-term post-event sport participation (*β* = 0.08, p < 0.005, SD = 0.043), providing support for H4. The results further indicated that perceived stress negatively moderated the relationship between sport attraction and near-term post-event sport participation (*β* = −0.100, *p* < 0.05, SD = 0.046), thus supporting H5. Figure 2 illustrates the form of this interaction (lower slope = 0.329, higher slope = 0.153). Overall, these findings provide empirical support for the earlier discussion based on the PCM (Table 6).

**Table 5:** Collinearity statistics (VIF) and effect sizes (F^2^).

Path	VIF	F^2^
SA → SP	1.927	0.291
PS → SP	1.799	0.043
DI → SA	1.000	0.133
SA*PS → SP	1.096	0.041

**Table 6:** Assessment of the structural model.

Paths	Beta value	SD	CIBC (LL)	CIBC (LU)	*P* value	Supported or not supported
H1 DI → SP	0.192	0.058	0.068	0.295	0.001	Supported
H2 DI → SA	0.333	0.095	0.138	0.510	0.000	Supported
H3 SA → SP	0.241	0.043	0.154	0.308	0.000	Supported
H4 DI → SA → SP	0.08	0.027	0.036	0.144	0.003	Supported
H5 PS*SA → SP	−0.100	0.046	−0.178	−0.013	0.029	Supported

## 5 Discussion

### 5.1 Theoretical implications

This study examines the influence of destination image on tourist behavior in the context of sport event tourism, thereby advancing understanding of how sport events shape the subsequent behavioral outcomes. By integrating spillover effects and the psychological continuum model (PCM), this study empirically tests the relationships among destination image, sport attraction, near-term post-event sport participation, and perceived stress. Notably, given the relatively short measurement window, our investigation does not capture the full progressive continuum of the PCM; rather, it focuses specifically on the short-term behavioral impact immediately following the event, which nonetheless offers several theoretical contributions to understanding event-induced behavioral spillover.

First, building on Afshardoost and Eshaghi (2020), this research provides empirical evidence that destination image exerts a significant influence on tourist behavior in sport event tourism. Although prior studies have investigated how destination image relates to tourists’ behavioral intentions from various perspectives, robust evidence linking destination image to actual near-term post-event sport participation remains limited. Recent research (e.g., Kim and Kaplanidou, 2019) has begun to employ frameworks such as social exchange theory and the theory of planned behavior to explore connections between sport events and participation-related outcomes. Extending this line of inquiry, the present study clarifies the pathway from destination image to near-term post-event sport participation via sport attraction, thereby strengthening both theoretical explanation and empirical support. These results also highlight the value of future longitudinal designs that can more directly capture how destination image translates into near-term post-event participation over time.

Second, this study contributes to research on sport attraction and the psychological continuum model. The PCM emphasizes the role of attraction as a foundation for developing sport attachment and loyalty, yet empirical evidence testing this mechanism has remained relatively scarce. The findings demonstrate that a favorable destination image of a host city for sport events increases sport attraction among young tourists, which subsequently promotes near-term post-event sport participation. In doing so, the study provides empirical support for the PCM and clarifies sport attraction as an important psychological conduit linking destination perceptions to short term post-event sport participation, while acknowledging that our short measurement window captures the initial rather than the full progressive phase of the PCM continuum.

### 5.2 Practical implications

Understanding how destination image translates into near-term post-event sport participation offers practical implications for tourism marketers, destination managers, and sport organizations seeking to promote active lifestyles among young people. First, a positive destination image can enhance tourists’ attraction to sport. Accordingly, destinations should invest in maintaining and strengthening their perceived image, particularly by improving sport-related cognitive attributes, such as facility quality, event organization, accessibility, and safety, that shape tourists’ evaluations. Specifically, host cities and event organizers can optimize on-site venue layouts, refine event service processes, and release high-quality promotional videos featuring young participants to build a credible destination image. Local tourism authorities can integrate sport event resources with urban scenic spots and launch themed sport tourism routes to consolidate tourists’ perceptions. Cooperation with youth-oriented key opinion leaders to share authentic sport resource experiences can also enhance the persuasiveness of the destination image among young groups.

Second, sport attraction exerts a significant influence on tourists’ near-term post-event sport participation. However, increases in participation driven primarily by attraction may be difficult to sustain over time. Practitioners should therefore support tourists’ psychological progression toward deeper attachment, thereby fostering a more enduring emotional connection to sport. In practical terms, destination marketers can launch youth-oriented mass-participation sport events, such as campus sport leagues, urban mini-marathons, and amateur e-sports competitions, after the conclusion of major comprehensive games. Social media and short-video platforms, such as Douyin and Xiaohongshu, can be used to disseminate sport challenges, athlete highlights, and fitness-sharing content that help sustain young people’s interest in sport. In addition, cities can create a convenient environment for near-term post-event sport participation among young tourist groups by adding outdoor fitness stations near communities and campuses, opening stadiums and gymnasiums to the public at low cost or free of charge during non-competition periods, and providing free or discounted fitness coaching services for young people.

Third, the findings indicate that perceived stress moderates near-term post-event sport participation, highlighting the close interconnections among stress, physical activity, and health. Prior research suggests that stress is an important factor related to near-term post-event sport participation and health outcomes. Given the broader public health burden associated with elevated stress levels, policymakers may consider strengthening population-level stress monitoring and early intervention mechanisms, especially for young people, such as screening, education, and community-based support, to help individuals manage perceived stress and promote active lifestyles.

## 6 Conclusion

Sport event tourism has become an increasingly important setting for examining how tourism experiences extend into sport-related behavioral outcomes. Drawing on the spillover effect framework, the psychological continuum model (PCM), and prior literature, this study examined how destination image influences short term post-event sport participation following sport event tourism. The one-month interval between the two survey waves further positioned the study to capture short-term post-event behavioral responses. The findings provide evidence of a significant spillover process from destination image to sport attraction: a more favorable destination image enhances the perceived appeal of sport, which in turn increases tourists’ engagement in sport-related activities. Importantly, the transition from attraction to actual near-term post-event sport participation is negatively moderated by perceived stress. Specifically, as perceived stress increases, the positive effect of sport attraction on near-term post-event sport participation diminishes.

This study has several limitations. First, because the sample was drawn from the FISU World University Games and consisted predominantly of student participants, the generalizability of the findings may be limited. Future research should recruit more diverse demographic groups and examine a wider range of sport event contexts to enhance external validity. Longitudinal studies with more diverse non-student samples across different age and income levels are also needed to further enhance the external validity of the model. Second, logistical constraints restricted data collection to two time points, which may have limited the ability to capture longer-term effects. Additionally, our cross-sectional data limit our ability to track participants’ progression through the full PCM hierarchy (i.e., attachment, allegiance, and habit formation), which would require a multi-wave longitudinal design. To reduce potential bias associated with short-term or situational influences, future studies should adopt longitudinal designs. Such approaches are particularly well suited to investigating near-term post-event sport participation behaviors and may yield richer insights into temporal dynamics. Third, although this study identifies perceived stress as a moderator of near-term post-event sport participation outcomes, other potentially influential moderators were not examined. For example, Prins et al. (2010) reported that improved access to sport facilities is associated with increased near-term post-event sport participation. Accordingly, future research should investigate additional contextual and environmental factors as potential boundary conditions.

## Data Availability

The original contributions presented in the study are included in the article/Supplementary material, further inquiries can be directed to the corresponding author.
