# Supplementary material for: Sport event destination image promotes subsequent sport participation through sport attraction: the moderating role of perceived stress
Source: Front Psychol. 2026 Aug 3;17:1863652. doi: 10.3389/fpsyg.2026.1863652 (PMC13477875; doi:10.3389/fpsyg.2026.1863652)
Supplement: Supplementary file 1 [file Supplementary_file_1.docx]

**Appendix**

**Pre-test questionnaire**

Dear Participants,

Thank you for taking the time out of your busy schedule to complete this questionnaire. The survey consists of five sections.

The 31st Summer Universiade was successfully held in Chengdu from July 28, 2023. This questionnaire aims to investigate tourists’ perception of the destination image of the Universiade host city, as well as their sports participation behavior after the event. Please fill in the questionnaire according to your genuine personal thoughts and travel experience, and try not to skip any items.

All information you provide will be used solely for academic research purposes. No individual responses will be disclosed to any institution, and your personal information will be strictly kept confidential. Please feel free to answer all questions honestly.

We sincerely appreciate your kind support and participation!

Best regards

Section A

*2.Demographic characteristics*

Kindly mark your response in the most appropriate box.

Have you watched the Universiade live in action?

□yes	□no（to the end）

How long you stay around Chengdu?

□ Less than 3 days

□ 3-7 days

□ 7-14 days

□ More than 14 days

What types of travel companion do you select in this visitation?

□ Independent travel

□ Group travel with family/friends

□ Group package travel

□ Others

Gender

□ Male □ Female

Age

□ Below 18 years

□ 18-30 years

□ 31-40 years

□ 41-50 years

□ More than 50 years

Personal monthly income

□ Less than 3000 RMB

□ 3000-6000 RMB

□ 6001-9000 RMB

□ More than 9000 RMB

Marital Status

□ Married

□ Single

□ Divorced

□ Other

Level of Education

□ High school and below

□ Diploma

□ Bachelor’s degree

□ Master’s degree

□ Doctoral degree

Occupation

□ Student

□ Government and public institution employee

□ Self-employed person

□ Freelancer

□ Other

Section B

*2.Destination Image of Event Sport Tourism*

The following table shows the vocabulary that may be applicable to portray the image characteristics of Chengdu, the host city of the Universiade. Which of the following words do you think is more accurate to describe Chengdu? Please √ your answer.

| Affective image | | | | | | |
| --- | --- | --- | --- | --- | --- | --- |
| Distressing | 1 | 2 | 3 | 4 | 5 | Relaxing |
|  | | | | | | |
| Gloomy | 1 | 2 | 3 | 4 | 5 | Exciting |
|  | | | | | | |
| Sleepy | 1 | 2 | 3 | 4 | 5 | Arousing |
|  | | | | | | |
| Unpleasant | 1 | 2 | 3 | 4 | 5 | Pleasant |
| Cognitive image | | | | | | |
| Accessible | 1 | 2 | 3 | 4 | 5 | Isolated |
|  | | | | | | |
| Friendly | 1 | 2 | 3 | 4 | 5 | Cold |
|  | | | | | | |
| Lively | 1 | 2 | 3 | 4 | 5 | Stagnant |
|  | | | | | | |
| Interesting | 1 | 2 | 3 | 4 | 5 | Boring |
|  | | | | | | |
| Quiet | 1 | 2 | 3 | 4 | 5 | Noisy |
|  | | | | | | |
| Overcrowded | 1 | 2 | 3 | 4 | 5 | Sparse |

Ekinci, Y., & Hosany, S. (2006). Destination personality: An application of brand personality to tourism destinations. Journal of travel research, 45(2), 127-139.

Section C

*3.sport attraction*

First, the following questions describe how attractive sports are to you, and please think about your thoughts about participating in sports in real life. Please circle your answer according to your actual situation. The scoring is shown as follows:**1=Strongly Disagree (SD), 2=Disagree (D), 3=Neutral (N), 4=Agree (A), 5=Strongly Agree (SA)**.

|  | Strongly Disagree | Disagree | Neutral | Agree | Strongly Agree |
| --- | --- | --- | --- | --- | --- |
| I feel that participating in sport is very important to me. | 1 | 2 | 3 | 4 | 5 |
| I feel that sport offers me relaxation when pressures build up. | 1 | 2 | 3 | 4 | 5 |
| I think participating in sport is one of the most satisfying things I do. | 1 | 2 | 3 | 4 | 5 |
| I feel that I really enjoy participating or watching in sport. | 1 | 2 | 3 | 4 | 5 |

Kyle, G. T., & Mowen, A. J. (2005). An examination of the leisure involvement—Agency commitment relationship. Journal of Leisure Research, 37(3), 342-363.

Section D

4. *Perceived stress*

Do you have the following behaviors in your life? Please circle it. The scoring is shown below:**1=Strongly Disagree (SD), 2=Disagree (D), 3=Neutral (N), 4=Agree (A), 5=Strongly Agree (SA)**

|  | Strongly Disagree | Disagree | Neutral | Agree | Strongly Agree |
| --- | --- | --- | --- | --- | --- |
| 1. In the last month, how often have you been upset because of  something that happened unexpectedly | 1 | 2 | 3 | 4 | 5 |
| 2. In the last month, how often have you felt that you were unable to  control the important things in your life? | 1 | 2 | 3 | 4 | 5 |
| 3. In the last month, how often have you felt nervous and 'stressed”? | 1 | 2 | 3 | 4 | 5 |
| 4. In the last month, how often have you felt confident about your  ability to handle your personal problems? | 1 | 2 | 3 | 4 | 5 |
| 5. In the last month, how often have you felt that things were going  your way? | 1 | 2 | 3 | 4 | 5 |
| 6. In the last month, how often have you found that you could not cope  with all the things that you had to do? | 1 | 2 | 3 | 4 | 5 |
| 7. In the last month, how often have you been able to control irritations  in your life? | 1 | 2 | 3 | 4 | 5 |
| 8. In the last month, how often have you felt that you were on top of  things? | 1 | 2 | 3 | 4 | 5 |
| 9. In the last month, how often have you been angered because of  things that were outside of your control? | 1 | 2 | 3 | 4 | 5 |
| 10. In the last month, how often have you felt difficulties were piling up  so high that you could not overcome them? | 1 | 2 | 3 | 4 | 5 |

**Cohen, S. (1994). Perceived Stress Scale. Mind Garden. Inc. Retrieved from www. mindgarden. com.**

Section E

5. *Sport participation*

To what extent do you agree with the following sports-related descriptions? Please circle your answer. The scoring is shown below:**1 = Very few, 2 = Few, 3 = Moderate, 4 = Many, 5 = Very many.**

|  | Very few | few | Moderate | many | Very many |
| --- | --- | --- | --- | --- | --- |
| Behavior participation | | | | | |
| How many times do you engage in physical activities per month (e.g., walking, radio calisthenics, gateball, volleyball, table tennis, jogging, tai chi, cycling, running, badminton, basketball, tennis, football, track and field, aerobics, swimming, yoga, etc.)? | Less than once per month | 1–2 times per month | 3–4 times per month | 5–6 times per month | At least 7 times per month |
| I talk a lot about sports. | 1 | 2 | 3 | 4 | 5 |
| I often watch sports-related videos or watch sports live. | 1 | 2 | 3 | 4 | 5 |
| I often read sports-related information. | 1 | 2 | 3 | 4 | 5 |
| I subscribe to sports-related accounts, bloggers or others. | 1 | 2 | 3 | 4 | 5 |
| Affective participation | | | | | |
| Sports are a way for me to relax. | 1 | 2 | 3 | 4 | 5 |
| Sports are a waste of time. | 1 | 2 | 3 | 4 | 5 |
| I receive little satisfaction from sports. | 1 | 2 | 3 | 4 | 5 |
| Cognitive participation | | | | | |
| Please select one sport you are most familiar with and describe it with at least three keywords.  Options include: walking, radio calisthenics, frisbee, volleyball, table tennis, jogging, tai chi, cycling, running, badminton, basketball, tennis, football, track running, structured aerobics, swimming, yoga, etc. | | | | | |

Wanat, S. (1983). Book Review: Social Aspects of Sport: ELDON E. SNYDER, ELMER A. SPREITZER: SOCIAL ASPECTS OF SPORT, Prentice-Hall, Inc., Englewood Cliffs, New Jersey, 1983, 350 pp. International Review of Sport Sociology, 18(3), 113-115.

Thank you very much

**Formal Questionnaire**

Dear Participants,

Thank you for taking the time out of your busy schedule to complete this questionnaire. The survey consists of five sections.

The 31st Summer Universiade was successfully held in Chengdu from July 28, 2023. This questionnaire aims to investigate tourists’ perception of the destination image of the Universiade host city, as well as their sports participation behavior after the event. Please fill in the questionnaire according to your genuine personal thoughts and travel experience, and try not to skip any items.

All information you provide will be used solely for academic research purposes. No individual responses will be disclosed to any institution, and your personal information will be strictly kept confidential. Please feel free to answer all questions honestly.

We sincerely appreciate your kind support and participation!

Best regards

Kindly mark your response in the most appropriate box.

Have you watched the Universiade live in action?

□yes	□no（to the end）

How long you stay around Chengdu?

□ Less than 3 days

□ 3-7 days

□ 7-14 days

□ More than 14 days

What types of travel companion do you select in this visitation?

□ Independent travel

□ Group travel with family/friends

□ Group package travel

□ Others

Gender

□ Male □ Female

Age

□ Below 18 years

□ 18-30 years

□ 31-40 years

□ 41-50 years

□ More than 50 years

Personal monthly income

□ Less than 3000 RMB

□ 3000-6000 RMB

□ 6001-9000 RMB

□ More than 9000 RMB

Marital Status

□ Married

□ Single

□ Divorced

□ Other

Level of Education

□ High school and below

□ Diploma

□ Bachelor’s degree

□ Master’s degree

□ Doctoral degree

Occupation

□ Student

□ Government and public institution employee

□ Self-employed person

□ Freelancer

□ Other

Section B

*2.Destination Image of Event Sport Tourism*

The following table shows the vocabulary that may be applicable to portray the image characteristics of Chengdu, the host city of the Universiade. Which of the following words do you think is more accurate to describe Chengdu? Please √ your answer.

| Affective image | | | | | | |
| --- | --- | --- | --- | --- | --- | --- |
| Distressing | 1 | 2 | 3 | 4 | 5 | Relaxing |
|  | | | | | | |
| Gloomy | 1 | 2 | 3 | 4 | 5 | Exciting |
|  | | | | | | |
| Sleepy | 1 | 2 | 3 | 4 | 5 | Arousing |
|  | | | | | | |
| Unpleasant | 1 | 2 | 3 | 4 | 5 | Pleasant |
| Cognitive image | | | | | | |
| Accessible | 1 | 2 | 3 | 4 | 5 | Isolated |
|  | | | | | | |
| Friendly | 1 | 2 | 3 | 4 | 5 | Cold |
|  | | | | | | |
| Lively | 1 | 2 | 3 | 4 | 5 | Stagnant |
|  | | | | | | |
| Interesting | 1 | 2 | 3 | 4 | 5 | Boring |

Ekinci, Y., & Hosany, S. (2006). Destination personality: An application of brand personality to tourism destinations. Journal of travel research, 45(2), 127-139.

Section C

*3.sport attraction*

First, the following questions describe how attractive sports are to you, and please think about your thoughts about participating in sports in real life. Please circle your answer according to your actual situation. The scoring is shown as follows:**1=Strongly Disagree (SD), 2=Disagree (D), 3=Neutral (N), 4=Agree (A), 5=Strongly Agree (SA)**.

|  | Strongly Disagree | Disagree | Neutral | Agree | Strongly Agree |
| --- | --- | --- | --- | --- | --- |
| I feel that participating in sport is very important to me. | 1 | 2 | 3 | 4 | 5 |
| I feel that sport offers me relaxation when pressures build up. | 1 | 2 | 3 | 4 | 5 |
| I think participating in sport is one of the most satisfying things I do. | 1 | 2 | 3 | 4 | 5 |
| I feel that I really enjoy participating or watching in sport. | 1 | 2 | 3 | 4 | 5 |

Kyle, G. T., & Mowen, A. J. (2005). An examination of the leisure involvement—Agency commitment relationship. Journal of Leisure Research, 37(3), 342-363.

Section D 第四部分

4. *Perceived stress*

Do you have the following behaviors in your life? Please circle it. The scoring is shown below:**1=Strongly Disagree (SD), 2=Disagree (D), 3=Neutral (N), 4=Agree (A), 5=Strongly Agree (SA)**

|  | Strongly Disagree | Disagree | Neutral | Agree | Strongly Agree |
| --- | --- | --- | --- | --- | --- |
| In the last month, how often have you been upset because of something that happened unexpectedly | 1 | 2 | 3 | 4 | 5 |
| In the last month, how often have you felt that you were unable to control the important things in your life? | 1 | 2 | 3 | 4 | 5 |
| In the last month, how often have you felt nervous and 'stressed”? | 1 | 2 | 3 | 4 | 5 |
| In the last month, how often have you felt that things were going  your way? | 1 | 2 | 3 | 4 | 5 |
| In the last month, how often have you found that you could not cope with all the things that you had to do? | 1 | 2 | 3 | 4 | 5 |
| In the last month, how often have you been able to control irritations in your life? | 1 | 2 | 3 | 4 | 5 |
| In the last month, how often have you felt that you were on top of things? | 1 | 2 | 3 | 4 | 5 |
| In the last month, how often have you felt difficulties were piling up so high that you could not overcome them? | 1 | 2 | 3 | 4 | 5 |

Cohen, S. (1994). Perceived Stress Scale. Mind Garden. Inc. Retrieved from www. mindgarden. com.

Section E 第五部分

5. *Sport participation*

To what extent do you agree with the following sports-related descriptions? Please circle your answer. The scoring is shown below:**1 = Very few, 2 = Few, 3 = Moderate, 4 = Many, 5 = Very many.**

|  | Very few | Few | Moderate | Many | Very many |
| --- | --- | --- | --- | --- | --- |
| Behavior participation | | | | | |
| How many times do you engage in physical activities per month (e.g., walking, radio calisthenics, gateball, volleyball, table tennis, jogging, tai chi, cycling, running, badminton, basketball, tennis, football, track and field, aerobics, swimming, yoga, etc.)? | Less than once per month | 1–2 times per month | 3–4 times per month | 5–6 times per month | At least 7 times per month |
| I talk a lot about sports. | 1 | 2 | 3 | 4 | 5 |
| I often watch sports-related videos or watch sports live. | 1 | 2 | 3 | 4 | 5 |
| I often read sports-related information. | 1 | 2 | 3 | 4 | 5 |
| I subscribe to sports-related accounts, bloggers or others. | 1 | 2 | 3 | 4 | 5 |
| Affective participation | | | | | |
| Sports are a waste of time. | 1 | 2 | 3 | 4 | 5 |
| I receive little satisfaction from sports. | 1 | 2 | 3 | 4 | 5 |
| Cognitive participation | | | | | |
| Please select one sport you are most familiar with and describe it with at least three keywords.  Options include: walking, radio calisthenics, frisbee, volleyball, table tennis, jogging, tai chi, cycling, running, badminton, basketball, tennis, football, track running, structured aerobics, swimming, yoga, etc. | | | | | |

Wanat, S. (1983). Book Review: Social Aspects of Sport: ELDON E. SNYDER, ELMER A. SPREITZER: SOCIAL ASPECTS OF SPORT, Prentice-Hall, Inc., Englewood Cliffs, New Jersey, 1983, 350 pp. International Review of Sport Sociology, 18(3), 113-115.

Thank you very much
